# Raising the iron curtain: Lactate's secret role in oxidative stress defense

**DOI:** 10.1016/j.redox.2025.103754

**Published:** 2025-07-05

**Authors:** Astrid Hensel, Renáta Váraljai, Shirley K. Knauer

**Affiliations:** a Department of Dermatology, University Hospital Essen, D-45147, Essen, Germany; b Department of Dermatology, University Hospital Essen, West German Cancer Center, University Duisburg-Essen and the German Cancer Consortium (DKTK), D-45147, Essen, Germany; c Department of Molecular Biology II, Center for Medical Biotechnology (ZMB), University of Duisburg-Essen, D-45117, Essen, Germany

**Keywords:** Oxidative stress, Lactate, Ferroptosis, Fenton reaction, Labile iron pool

## Abstract

The hypothesis presented here is that certain cell types under oxidative stress, such as cancer cells, reprogram their metabolism to accumulate lactate, along with cytosolic Fe^2+^ within the labile iron pool, thereby establishing a metabolite-based H_2_O_2_ detoxification system. In this scenario, the Fenton reaction between Fe^2+^ and H_2_O_2_ generates hydroxyl radicals (HO•), which are subsequently scavenged by abundant lactate. Thus, lactate production may function as a protective, iron-dependent antioxidant mechanism, enabling cells to decompose H_2_O_2_ and prevent damage to crucial biomolecules. If this system is compromised, for instance by inadequate HO•-scavenging or impaired Fe^2+^ recycling, cells may become prone to ferroptosis.

## 1 Introduction

Oxidative stress occurs when reactive oxygen species (ROS) surpass a cell's antioxidant capacity. Among these, hydrogen peroxide (H_2_O_2_) is particularly critical due to its relative stability and high diffusibility compared to other ROS like hydroxyl radicals [1]. H_2_O_2_ can oxidize thiol groups and damage DNA even at distant intracellular sites, leading to apoptosis [[2], [3], [4], [5], [6]]. It is enzymatically degraded by catalase, glutathione peroxidase 1 (GPX1), and peroxiredoxins (Prx) [7]. While catalase is highly efficient, its confinement to peroxisomes limits its cytosolic availability. GPX1 and Prx serve as the main cytosolic defenses against H_2_O_2_ but may become saturated or transiently inactivated during high oxidative stress [8,9]. Major sources of H_2_O_2_ include innate immune cells, where NOX2 generates superoxide that is rapidly converted into H_2_O_2_ [[10], [11], [12], [13]], as well as electron leakage from the mitochondrial respiratory chain [1,11]. Although H_2_O_2_ functions as a signaling molecule at low concentrations [7], physiologically elevated concentrations are cytotoxic if antioxidant defenses are insufficient [5,6,8].

Oxidative stress is frequently accompanied by increased lactate production and dysregulated iron metabolism. A prime example of this association is cancer, in which metabolic reprogramming toward aerobic glycolysis leads to high lactate levels [[14], [15], [16]]. Simultaneously, many cancer cells expand their labile iron pool (LIP) [[17], [18], [19], [20], [21], [22], [23]], defined as the redox-active, loosely bound intracellular iron that can also act as a catalyst in the Fenton reaction [24]. Importantly, the LIP is not only elevated in proliferating cancer cells, which require iron for DNA synthesis and other proliferation-relevant enzymes, but also in non-proliferating quiescent cancer cells [25]. Additionally, intracellular iron and excess ROS drive ferroptosis, a non-apoptotic form of cell death [[26], [27], [28], [29]]. Ferroptosis is characterized by the lethal accumulation of lipid peroxides, driven by iron-catalyzed oxidative reactions that generate lipid peroxidation at levels exceeding the repair capacity of glutathione peroxidase 4 (GPX4), which specifically reduces lipid hydroperoxides [30].

**Fig. 1:**
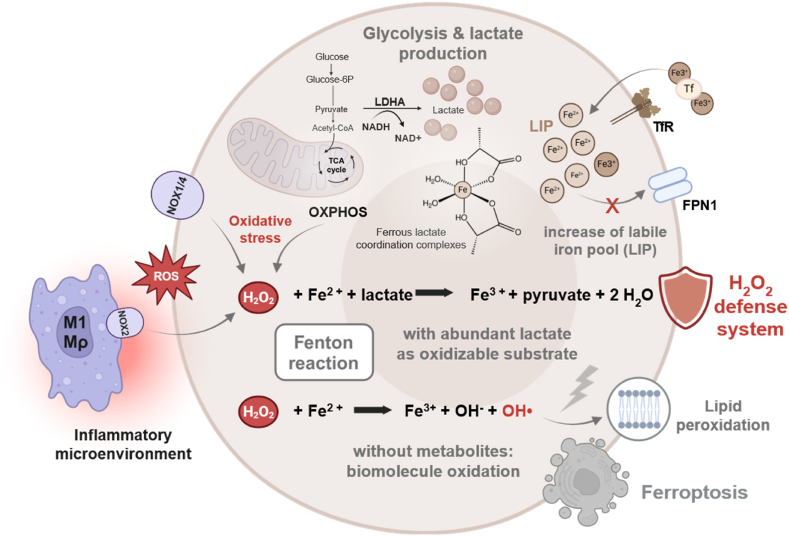
**The Warburg effect as a protective mechanism against oxidative Stress: Lactate and Fe**^**2+**^**in H**_**2**_**O**_**2**_**detoxification.** Oxidative stress arises when reactive oxygen species (ROS) surpass a cell's antioxidant capacity. ROS primarily originate from incomplete oxygen reduction, such as electron leakage in the mitochondrial respiratory chain. During inflammation, phagocytes like macrophages and neutrophils actively generate ROS through NOX2 (NADPH oxidase 2), while cancer cells themselves can contribute via NOX1 and NOX4, further increasing intracellular ROS levels. Superoxide, a prevalent ROS, is rapidly converted into H_2_O_2_ - a less reactive but highly diffusible molecule capable of damaging biomolecules. In cells with an expanded labile iron pool, Fe^2+^ reacts with H_2_O_2_ via the Fenton reaction, producing highly reactive hydroxyl radicals (HO•). However, before these radicals can inflict cellular damage, they are scavenged by lactate, which is simultaneously oxidized to pyruvate. At the site of hydroxyl radical formation, lactate - coordinated within iron complexes - serves as a preferential substrate for oxidation, protecting essential biomolecules from oxidative stress. When oxidizable metabolites are scarce or Fe^3+^ is not regenerated to Fe^2+^, the resulting hydroxyl and hydroperoxyl radicals drive biomolecular damage, particularly lipid peroxidation, ultimately triggering ferroptosis. Thus, lactate production via aerobic glycolysis may function as a non-enzymatic ROS defense strategy in cancer cells and other lactate-producing cell types, shielding them from oxidative stress-induced cell death. Created with Biorender.com.

In this hypothesis, we propose a mechanistic link between increased lactate production, elevated cytosolic Fe^2+^ levels, and heightened oxidative stress by introducing a previously overlooked role of lactate in H_2_O_2_ detoxification (Fig. 1).

## 2 Hypothesis: A lactate-iron system for non-enzymatic H_2_O_2_ detoxification

Several cell types, including cancer cells, activated macrophages, neutrophils, inflammatory synoviocytes and astrocytes, exhibit both increased lactate production and elevated labile iron pools (LIP) under specific conditions. Regarding lactate production, this metabolic reprogramming is exemplified in cancer cells by aerobic glycolysis, also known as the Warburg effect [14,15]. In this process, glucose is metabolized to pyruvate and subsequently reduced to lactate by lactate dehydrogenase A (LDHA), even in the presence of oxygen (Fig. 1). Similarly, other cell types generate lactate under both physiological and pathological conditions [[31], [32], [33], [34]]. While the precise advantages of lactate synthesis remain poorly understood, this metabolic adaptation likely serves functions beyond hypoxia tolerance. Notably, it may provide a crucial survival mechanism against a common cellular threat: oxidative stress [1,35].

Furthermore, the glycolytic phenotype is closely linked to elevated intracellular iron levels, a hallmark feature observed in many of these cell types [17,36,37]—including RAS-mutant cancers, where both glycolysis and intracellular iron levels are enhanced through activation of the MAPK/ERK signaling pathway [38,39]. Cancer cells, among others, enhance iron uptake by upregulating transferrin receptor 1 (TFRC) while simultaneously downregulating ferroportin, the only known iron exporter [17,18,28]. HIF-1α, an essential regulator active under both hypoxic conditions and oxidative stress, upregulates TFRC and LDHA, promoting increased iron uptake and enhanced glycolytic activity [[40], [41], [42]]. Additionally, reduced ferritin levels can lead to the mobilization of stored iron into the cytosol, thereby increasing the LIP.

Under inflammatory conditions, the iron-regulating hormone hepcidin exacerbates this effect by promoting ferroportin degradation and preventing iron export, thereby contributing to the further elevation of the LIP [43]. The resulting accumulation of bioavailable Fe^2+^ fuels the Fenton reaction, in which Fe^2+^ reacts with H_2_O_2_ to generate highly reactive hydroxyl radicals (HO•) [44,45]. These radicals are significantly more potent oxidizing agents than H_2_O_2_ itself. While the Fenton reaction is typically viewed as harmful due to its role in producing ROS that can drive lipid peroxidation and ferroptosis [24,26,45], this interpretation overlooks the reliance of this reaction on H_2_O_2_ as a reactant.

Given that cancer cells actively elevate their LIP, an alternative perspective emerges: Fe^2+^-driven HO• formation may, paradoxically, serve a protective purpose - but only if abundant lactate is available to intercept these radicals. In this scenario, lactate and iron collaborate to detoxify H_2_O_2_ via the Fenton reaction, with HO• preferentially oxidizing lactate due to its high cytosolic availability and spatial coordination within iron-lactate complexes. By acting as a readily available oxidizable target, lactate scavenges HO• radicals at their site of formation, preventing uncontrolled oxidation of critical biomolecules such as enzymes, lipids, and DNA. Thus, the cytosolic accumulation of Fe^2+^ and lactate forms an antioxidant buffer, enabling cancer cells and other lactate-producing cells to mitigate oxidative stress - an adaptive strategy potentially shared across a range of cellular contexts.

## 3 Public dataset analysis: Further evidence supporting the H_2_O_2_-lactate-iron axis

**Fig. 2:**
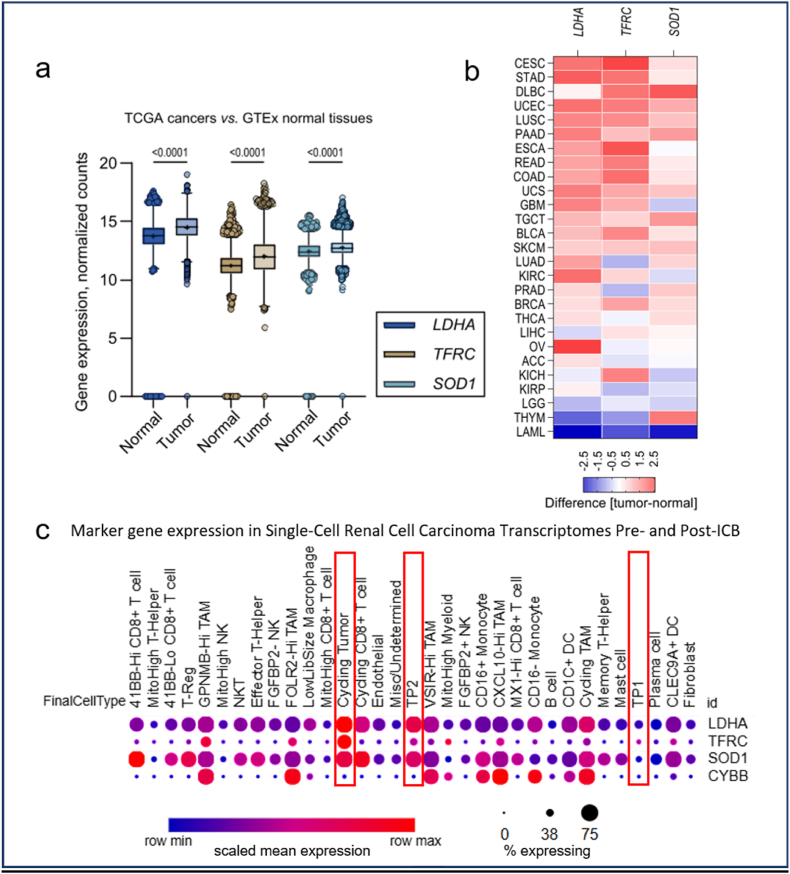
**Concerted upregulation of LDHA, TFRC, and SOD1 indicates enhanced lactate production, labile iron pool, and oxidative stress in cancer.** (A) Box and whiskers plots depicting gene expression levels of LDHA, TFRC, and SOD1 in The Cancer Genome Atlas (TCGA, n = 9807 tumor tissues) and the reference tissue GTEx datasets (n = 7414 normal tissue samples). P values are from 1-way ANOVA with Holm-Šídák's correction for multiple comparisons. (B) Heat map showing the fold difference of expression of LDHA, TFRC, and SOD1 between TCGA tumor and GTEx normal datasets matched according to organ site. (C) Dot plot analysis of single-cell transcriptomes from human renal cell carcinoma pre- and post-immunotherapy, comparing expression levels of *LDHA*, *TFRC*, *SOD1*, and *CYBB* (NOX2) across distinct cell populations. Tumor cell subpopulations are highlighted by red rectangles. Data were obtained from the Single Cell Portal [66] (https://singlecell.broadinstitute.org/single_cell), based on the study *Tumor and immune reprogramming during immunotherapy in advanced renal cell carcinoma* [46].

Fig. 2 demonstrates the correlation between lactate production, increased LIP, and H_2_O_2_-mediated oxidative stress in cancer, as evidenced by the increased transcription of LDHA, TFRC, and cytosolic superoxide dismutase (SOD1).

Bulk RNA-Seq analysis (Fig. 2A & B) reveals a consistent and statistically significant upregulation of these genes across 33 TCGA tumor types compared to GTEx normal tissues (Fig. 2A), with further resolution into 27 cancer entities (Fig. 2B and Supplemental Fig. S1). However, TCGA bulk RNA-Seq data integrates signals from tumor, stromal, and immune cells, thereby restricting detailed cell-type-specific interpretation. As an example, single-cell RNA-Seq (scRNA-Seq) data from renal cell carcinoma [46] provides cell-type resolution (Fig. 2C), identifying distinct tumor subpopulations: 'Cycling Tumor' and 'TP2' tumor cells markedly co-upregulate TFRC, LDHA, and SOD1, suggesting an adaptive response to oxidative stress, potentially influenced by tumor-associated macrophages (TAMs), which show particularly high NOX2 (CYBB) expression (Fig. 2C). In contrast, 'TP1' tumor cells display an opposing transcriptional profile, suggesting metabolic heterogeneity and distinct immune interactions within the tumor microenvironment.

Notably, the 'Cycling tumor' and 'TP2' cancer cell subpopulations do not show increased levels of enzymatic H_2_O_2_ detoxification systems (Supplemental Fig. S2) despite experiencing H_2_O_2_ stress - further evidence that H_2_O_2_ detoxification is not solely dependent on enzymatic mechanisms.

## 4 Mechanistic basis: The Fenton reaction as a detoxification pathway

Several studies have described potential antioxidant roles of lactate [47,48]. However, due to its relatively high redox potential, lactate alone is not considered a classical antioxidant and cannot directly reduce H_2_O_2_. The novelty of our perspective lies in the idea that lactate can exert a H_2_O_2_-detoxifying effect only when functionally coupled with available Fe^2+^, which first converts H_2_O_2_ into reactive hydroxyl radicals via the Fenton reaction.

**Scheme 1:**
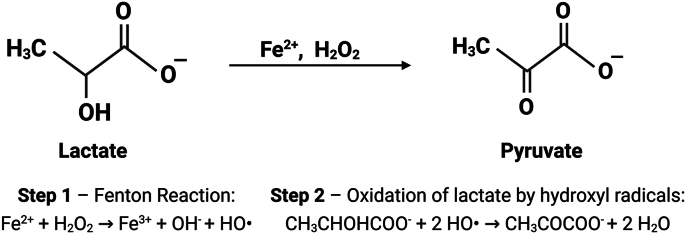
**The Fenton reaction.** The reaction proceeds through the generation of hydroxyl radicals (HO•), formed by the interaction of H_2_O_2_ and Fe^2+^. Within a lactate molecule, the proximity of the hydroxyl and carboxyl groups makes the α-C-H bond particularly vulnerable to attack by HO•. The hydroxyl radical abstracts a hydrogen atom from the α-C-H bond, forming a radical intermediate (CH_3_C•OHCOOH). A second HO• then abstracts a hydrogen atom from the hydroxyl group of this intermediate, resulting in the formation of a carbonyl group and ultimately yielding pyruvate. Furthermore, lactate can bind Fe^2+^ to form ferrous lactate complexes, which stabilize Fe^2+^ in a reactive form and facilitate the reaction [50].

The Fenton reaction involves Fe^2+^ catalyzing the oxidation of organic substrates by H_2_O_2_ under acidic conditions (Scheme 1). In 1894, Fenton demonstrated that Fe^2+^ could catalyze the oxidation of tartaric acid by H_2_O_2_ [49]. Similarly, lactate, also a hydroxycarboxylic acid, undergoes a reaction with H_2_O_2_ and Fe^2+^ to form pyruvate [50]. Mechanistically, the Fenton reaction occurs through the formation of hydroxyl radicals when H_2_O_2_ reacts with Fe^2+^ [44]. These highly reactive radicals possess strong oxidative potential and will react with the first available substrate they encounter. In cells with high lactate concentrations, the hydroxyl radicals are more likely to interact with and oxidize lactate rather than other critical biomolecules. For example, in situ generated H_2_O_2_ in a pyrite suspension has been shown to oxidize lactate via hydroxyl radicals [50], confirming its role as a HO• target under Fenton-like conditions.

Bioavailable Fe^2+^ ions in the LIP do not exist freely but are incorporated into coordination complexes with low-molecular-weight ligands that remain loosely associated, including peptides and carboxylates [51,52]. Under conditions of high lactate concentrations, lactate itself may become the predominant ligand, positioning it close to the site of hydroxyl radical formation [50]. This proximity could facilitate lactate oxidation and enable it to act as a scavenger for the reactive hydroxyl radical intermediates in the Fenton reaction. Given the millimolar abundance of lactate and its ability to weakly coordinate Fe^2+^ (log β_1_ ≈ −1.3, according to NIST SRD 46 [53]), transient Fe^2+^-lactate complexes are likely to form within the cytosolic labile iron pool. These labile complexes can maintain iron in a redox-active state and thereby enable Fenton-type ROS reactivity [54].

The same reaction can also occur extracellularly, such as in the tumor microenvironment (TME) or in inflamed tissues. In these contexts, the combination of accumulated lactate and released Fe^2+^ facilitates the capture and detoxification of H_2_O_2_ through the Fenton reaction, contributing to the regulation of oxidative stress and protecting surrounding cells and biomolecules from ROS-induced damage.

**Fig. 3:**
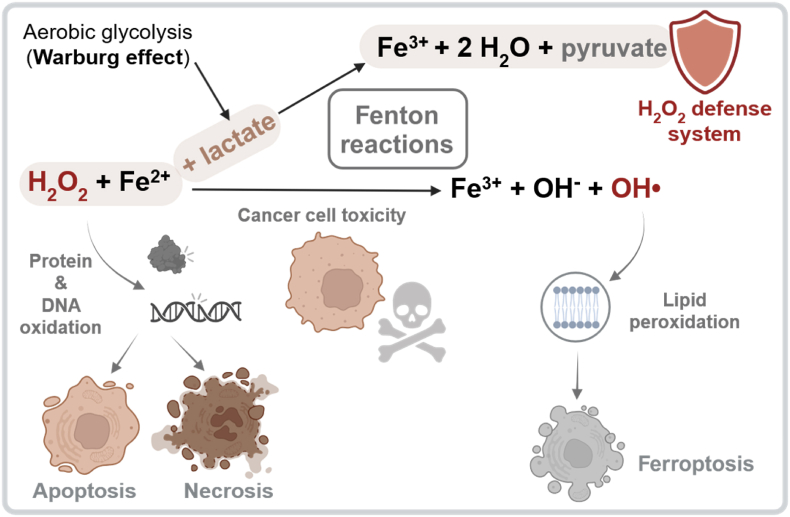
**Ferrous iron (Fe**^**2+**^**) and lactate cooperate in Fenton-based H**_**2**_**O**_**2**_**detoxification.** A protein-free H_2_O_2_ scavenging system operates with soluble Fe^2+^ and enriched oxidizable metabolites, such as lactate, in the cytosol to facilitate H_2_O_2_ decomposition via the Fenton reaction. In this process, Fe^2+^ transfers an electron to H_2_O_2_, generating highly reactive hydroxyl radicals (HO•). These radicals react at diffusion-controlled rates, meaning they interact immediately with the first available substrate. At elevated cytosolic concentrations, lactate acts as a hydroxyl radical scavenger, protecting essential biomolecules from oxidative damage. In cells with a low labile iron pool (LIP) and low lactate production, excess H_2_O_2_ can oxidize proteins and DNA, leading to apoptosis or necrosis [2]. Conversely, if both H_2_O_2_ and cytosolic Fe^2+^ are abundant but there are insufficient oxidizable metabolites such as lactate, the generated hydroxyl radicals preferentially oxidize lipids, thereby triggering ferroptosis. Created with Biorender.com.

The Fenton reaction is also utilized in wastewater treatment to degrade organic contaminants through the addition of hydrogen peroxide and iron. In contrast, lactate-producing cells may exploit this reaction to decompose harmful H_2_O_2_ by using iron as a catalyst and lactate as a metabolite that can be oxidized without causing harm to the cell (Fig. 3).

Unlike in classical iron-dependent enzymatic systems, where iron functions as a tightly bound cofactor within proteins that catalyze redox reactions, the described mechanism relies on catalysis by soluble Fe^2+^. This points to a non-enzymatic antioxidant strategy, in which free iron and abundant lactate together detoxify H_2_O_2_ without requiring the upregulation of antioxidant enzymes. This idea is supported by non-enzymatic, iron-catalyzed reactions observed under physiologically relevant conditions in core pathways like glycolysis and the pentose phosphate pathway [55], suggesting that such chemistry may represent a latent layer of metabolic regulation.

## 5 “Rescue mode oxidation”: Coupling H_2_O_2_ detoxification to metabolite oxidation

Fe^2+^ ions in the cytosol catalyze the conversion of harmful H_2_O_2_ into hydroxyl radicals (HO•) through the Fenton reaction, driving the targeted oxidation of enriched lactate. If lactate can act as a sacrificial substrate for hydroxyl radicals under Fenton-like conditions, it follows that other oxidizable metabolites—such as the TCA cycle intermediates citrate and α-ketoglutarate—could also contribute to H_2_O_2_ detoxification upon export from mitochondria into the cytosol, where they become accessible to Fenton chemistry. This shifts metabolite oxidation from the mitochondria to the cytosol, linking H_2_O_2_ clearance to the controlled oxidation of metabolites. The oncometabolite D-2-hydroxyglutarate (D-2HG) may likewise undergo Fenton-mediated oxidation, as it reacts readily with hydroxyl radicals, serving as a redox-sensitive metabolite that regenerates α-ketoglutarate.

Unlike the mitochondrial respiratory chain, where O_2_ acts as the final electron acceptor, this cytosolic process uses H_2_O_2_. Here, lactate or other cytosolic metabolites are oxidized instead of reduction equivalents like NADH or FADH_2_. However, ATP continues to be produced during glycolysis, and NAD^+^ is regenerated during pyruvate-to-lactate conversion, maintaining metabolic flux.

This “rescue mode” oxidation provides a flexible fallback strategy under oxidative stress, sustaining redox homeostasis and metabolic activity while protecting essential biomolecules from oxidative damage. Under conditions of excess H_2_O_2_, lactate can be reoxidized to pyruvate via a Fenton-driven reaction that simultaneously reduces H_2_O_2_. The resulting pyruvate can re-enter central metabolism, supporting anabolic processes or fueling the TCA cycle. Cells may even promote endogenous H_2_O_2_ production to fuel this alternative oxidation route and gain a selective advantage under high-ROS conditions [56].

Notably, this metabolite-based redox buffering may extend beyond cancer metabolism. Emerging evidence indicates that cytosolic accumulation of TCA cycle intermediates, such as citrate and α-ketoglutarate, can confer protective effects under conditions of inflammation, oxidative stress, or cellular degeneration [[57], [58], [59], [60], [61]]. This broader relevance highlights the potential of Fenton-linked metabolite oxidation as a conserved stress response mechanism across diverse cell types.

## 6 Recycling of Fe^3+^ to Fe^2+^: Preventing ferroptosis

**Scheme 2:**
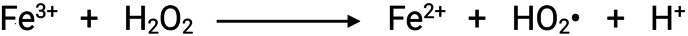
Fe^3+^-mediated Fenton Reaction generates hydroperoxyl radicals, fueling radical chain reactions and oxidative stress.

Recycling Fe^3+^ to Fe^2+^ is a critical and potentially rate-limiting step in the Fenton process, also playing a central role in Fenton-based wastewater treatment [62]. When Fe^2+^ levels are insufficient, Fe^3+^ reacts with H_2_O_2_ instead (Scheme 2), generating hydroperoxyl radicals (HOO•), which trigger uncontrolled radical chain reactions, ultimately leading to cellular damage. For example, HOO• extracts hydrogen from polyunsaturated fatty acids (PUFAs) in lipid membranes, initiating lipid peroxidation and potentially causing ferroptosis. While HOO• can also oxidize metabolites like lactate, this reaction further propagates the radical chain, regenerating H_2_O_2_ and intensifying oxidative stress rather than alleviating it.

In the cytosol, glutathione (GSH) plays a crucial role as a reductant of Fe^3+^, owing to its high intracellular concentration and potent reducing capacity through its thiol group (-SH) [52]. In addition to glutathione, cysteine can also reduce Fe^3+^, either independently or as a constituent of glutathione. Vitamin C (ascorbate), although mainly active in extracellular environments, can further reduce Fe^3+^ if present. The availability of cysteine is regulated by the cystine/glutamate antiporter (system xc^−^), which imports cystine into the cell, where it is reduced to cysteine through thioredoxin and NADPH-dependent pathways. When system xc^−^ is disrupted, such as by the compound erastin, intracellular cysteine levels drop [26], hindering Fe^2+^ regeneration and triggering ferroptosis - even in the presence of iron and oxidizable metabolites like lactate.

## 7 Therapeutic implications of the proposed lactate-iron-H_2_O_2_ system

The proposed lactate-iron-H_2_O_2_ axis highlights a previously overlooked mechanism driven by high cytosolic H_2_O_2_, Fe^2+^, and lactate concentrations, where the Fenton reaction generates hydroxyl radicals that primarily oxidize lactate, facilitating H_2_O_2_ detoxification. This concept has significant therapeutic potential (Table 1), particularly in cancer treatment, by targeting tumor cells’ reliance on lactate and bioavailable iron(II) as components of their antioxidant defense.

**Table 1:** Potential interventions to target the lactate-iron-H_2_O_2_ axis.

Targeting Cancer Cells by Inhibiting the Lactate-Fe^2+^ System:	Enhancing the H_2_O_2_ Detoxification System in Non-Cancerous Contexts:
**Iron Chelators**	**Reducing Agents/Antioxidants**

**Mechanism:** Iron chelators like DFO bind extracellular Fe^3+^ in the blood, reducing cellular iron uptake and intracellular Fe^2+^ levels & Fenton reaction. **Example:** Deferoxamine (DFO)	**Mechanism:** Enhancing cellular redox balance by replenishing glutathione (GSH) or cysteine for Fe^3+^ reduction, thereby regenerating Fe^2+^ for Fenton-based lactate oxidation. In addition, dietary uptake of further antioxidants—such as the reduced form of coenzyme Q10 (ubiquinol)— can act as ROS scavengers. **Examples:** NAC, ascorbate, Q10 (ubiquinol)

**Lactate Dehydrogenase Inhibitors (LDHA Inhibitors)**	**Iron Modulators**

***Mechanism*:** Blocking the conversion of pyruvate to lactate and reducing intracellular lactate levels. ***Examples*:** FX11, GSK2837808A	***Mechanism*:** Maintaining optimal Fe^2+^ levels to support the Fenton-based detoxification system without tipping into toxic Fe^3+^ accumulation. **Examples:** Controlled ferritinophagy (regulated ferritin degradation to release Fe^2+^), ferroportin stabilization (to prevent excessive iron retention), and modulation of transferrin receptor (TfR1) expression

**ROSModulators**	**Metabolic Modulators**

***Mechanism*:** Enhancing the H_2_O_2_ production selectively in cancer cells. Combined with reduced Fe^2+^ availability, this could trigger ferroptosis or apoptosis. ***Examples*:** β-lapachone, artemisinin	***Mechanism*:** Enhancing the glycolytic flux to increase lactate availability for H_2_O_2_ detoxification. ***Examples*:** Physical exercise, Lactated Ringer's solution (i.v.), FG-4592 (Roxadustat), Metformin Physical exercise generates lactate, which is then distributed to other tissues via the bloodstream. Roxadustat, by mimicking hypoxia, stimulates glycolysis and lactate production, while Metformin, by inhibiting the mitochondrial ETC complex I, similarly promotes glycolysis.

Conversely, in diseases where this detoxification system may offer protective benefits, interventions could enhance its function to reduce oxidative damage. One such approach involves N-acetylcysteine (NAC), a widely studied therapeutic antioxidant for various conditions, including neurodegenerative disorders [63], myocardial infarction [64], and multiple sclerosis (MS) [65]. As a cysteine precursor, NAC replenishes intracellular cysteine levels, supporting glutathione (GSH) synthesis and Fe^2+^ regeneration. By maintaining Fe^2+^ availability, NAC sustains the proposed antioxidant system involving Fe^2+^ and lactate, preventing the accumulation of hydroperoxyl radicals (HOO•), mitigating lipid peroxidation, and reducing oxidative damage. Consequently, NAC may act as a potential ferroptosis inhibitor by stabilizing redox homeostasis and enhancing ROS detoxification. In addition to pharmacological interventions, physical activity itself may enhance the lactate-based antioxidant buffering system in tissues and blood. During exercise, elevated lactate levels may strengthen this defense by scavenging H_2_O_2_, reducing oxidative stress, and shielding biomolecules from damage, further supporting the role of lactate as a key player in oxidative stress management. This mechanism could contribute to the well-documented protective effects of regular exercise against chronic diseases such as cardiovascular conditions, type 2 diabetes, neurodegenerative disorders (e.g., Alzheimer's), and chronic inflammation. Given that some non-malignant glycolytic cells depend on Fe^2+^-metabolite complexes to maintain redox homeostasis, the indiscriminate use of iron chelators or ROS-modulating agents may inadvertently trigger off-target cell death, either through apoptosis or, in iron-rich environments, ferroptosis. Therapeutic approaches should therefore be designed to selectively disrupt this redox axis in cancer cells while safeguarding protective buffering mechanisms in healthy tissues.

## 8 Conclusions and outlook

We propose a redox-based, non-enzymatic system for H_2_O_2_ detoxification that operates through the interaction of elevated cytosolic Fe^2+^ and abundant lactate or other oxidizable metabolites. According to this hypothesis, hydroxyl radicals (HO•), generated via the Fenton reaction from H_2_O_2_ and Fe^2+^, are immediately intercepted by lactate, which acts as a preferential oxidation target. This process prevents ROS-induced damage to essential biomolecules and may represent a metabolite-centered antioxidant defense mechanism, particularly relevant in cancer cells and other stress-adapted cell types.

To experimentally validate this hypothesis, future studies should aim to distinguish non-enzymatic lactate oxidation from enzymatic LDHB-mediated activity. This can be achieved through a multi-faceted experimental approach, including LDHB inhibition or knockout, modulation of cofactors, and the application of competing hydroxyl radical scavengers such as Tempol or GSH. Isotope tracing experiments under defined H_2_O_2_ and Fe^2+^ conditions, coupled with these interventions, could provide direct evidence that lactate is converted to pyruvate via a non-enzymatic, iron- and peroxide-dependent pathway.

Additionally, biochemical assays measuring ROS damage markers in response to lactate modulation, iron chelation, or ferroptosis induction could clarify whether lactate indeed serves as a sacrificial redox buffer. Real-time detection of hydroxyl radical activity or redox-probe-based imaging may further substantiate the involvement of this mechanism.

If verified, the proposed H_2_O_2_-lactate-iron axis could have far-reaching translational implications. In cancer therapy, selectively targeting this system, via LDHA inhibition and modulation of iron availability, may sensitize tumor cells to ROS-induced cytotoxicity.

Beyond cancer, it is important to investigate whether the proposed lactate–iron axis contributes to redox regulation in other physiological or pathological contexts characterized by oxidative stress. For example, the potential interplay between lactate production in adipocytes and insulin resistance in type 2 diabetes may reflect a role for this mechanism in systemic antioxidant defense. Additionally, given that Alzheimer's disease-related proteins, amyloid-β and tau, can bind iron in various forms, it is worth exploring whether these proteins are also susceptible to iron-dependent Fenton oxidation.

Exploring these mechanisms may help uncover non-enzymatic, metabolite-driven redox principles that potentially underlie diverse and seemingly unrelated disease contexts.

## CRediT authorship contribution statement

Astrid Hensel: Conceptualization (primary hypothesis development), Writing – original draft, Visualization. Shirley K. Knauer: Conceptualization (contributed to the conceptual development of the hypothesis), Writing – original draft, Visualization. Renáta Váraljai: Formal analysis (analyzed the expression of glycolysis-, iron-uptake-, and oxidative stress-related genes in cancer and normal tissues using public datasets), Data curation. All authors contributed to the review and editing of the manuscript.

## Declaration of competing interest

The authors declare that they have no known competing financial interests or personal relationships that could have appeared to influence the work reported in this paper.

## Data Availability

No new data were generated for this article. Publicly available datasets were reanalyzed in the context of the presented hypothesis.
