# Supplementary material for: Raising the iron curtain: Lactate's secret role in oxidative stress defense
Source: Redox Biol. 2025 Jul 5;85:103754. doi: 10.1016/j.redox.2025.103754 (PMC12281059; doi:10.1016/j.redox.2025.103754)
Supplement: Multimedia component 1 [file mmc1.pdf]

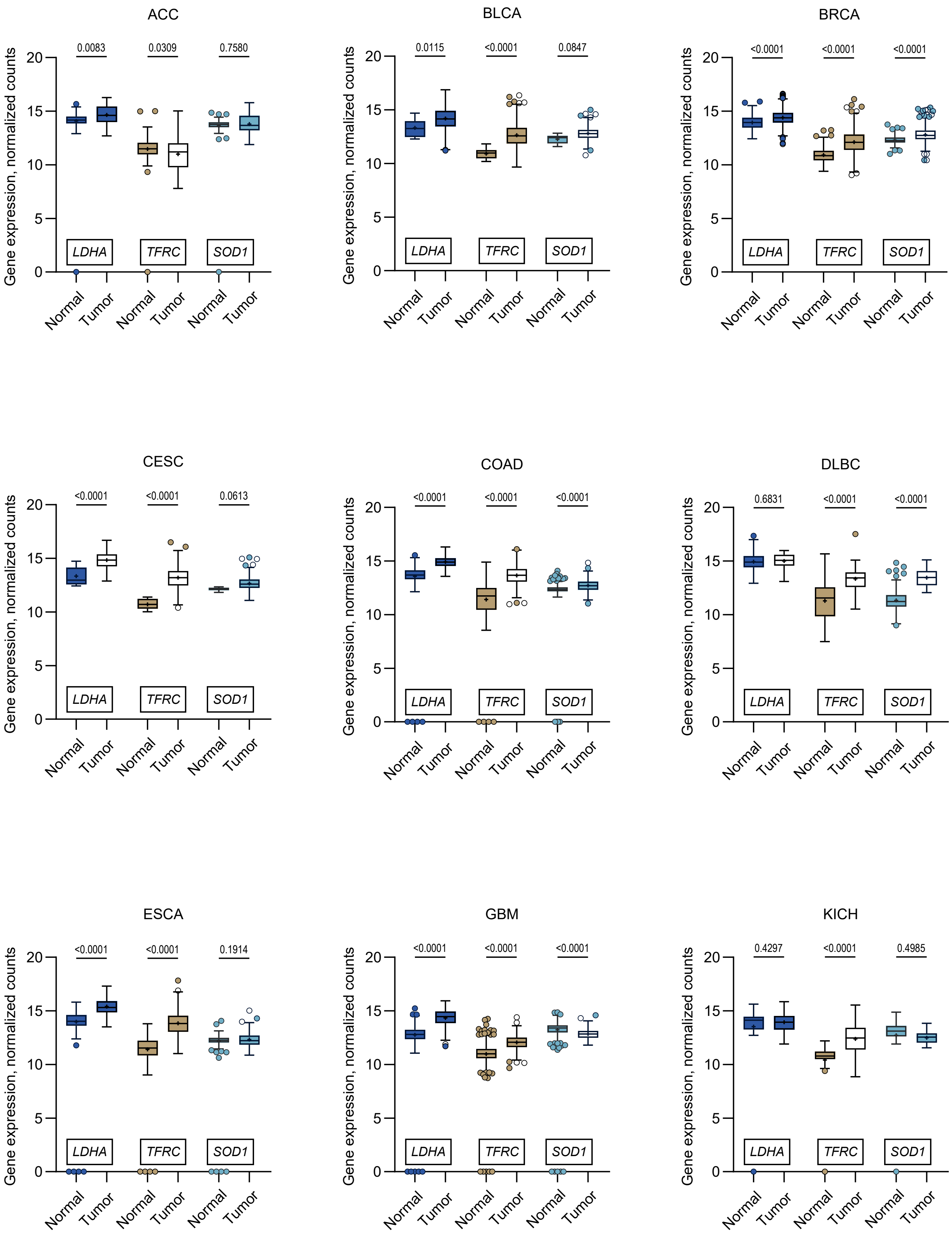

ACC
0.0083
0.0309
0.7580
BLCA
0.0115
<0.0001
0.0847
BRCA
<0.0001
<0.0001
<0.0001
CESC
<0.0001
<0.0001
0.0613
COAD
<0.0001
<0.0001
<0.0001
DLBC
0.6831
<0.0001
<0.0001
ESCA
<0.0001
<0.0001
0.1914
GBM
<0.0001
<0.0001
<0.0001
KICH
0.4297
<0.0001
0.4985
Gene expression, normalized counts
0
5
10
15
20
LDHA
TFRC
SOD1
Normal
Tumor

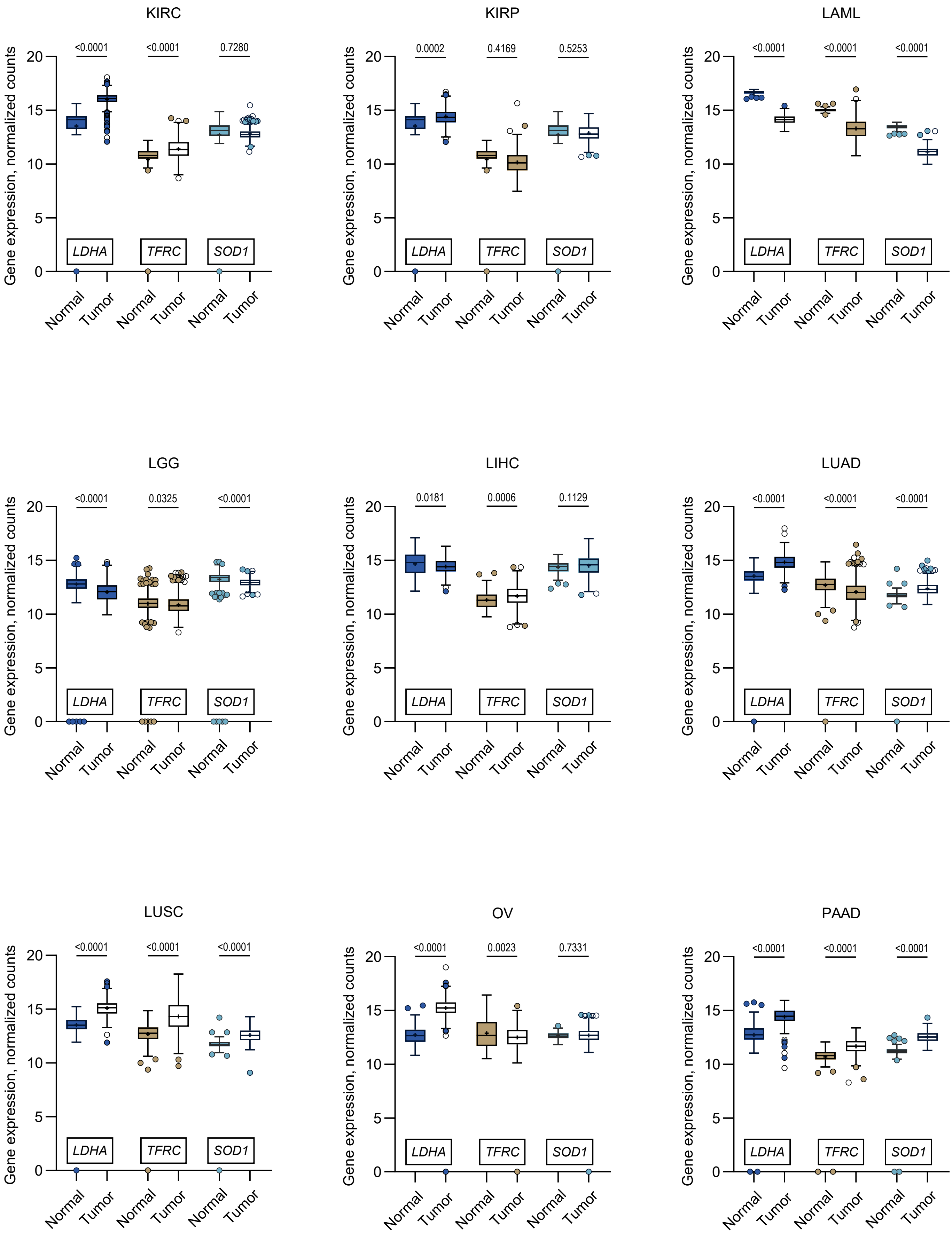
KIRC
<0.0001
<0.0001
0.7280
KIRP
0.0002
0.4169
0.5253
LAML
<0.0001
<0.0001
<0.0001
LGG
<0.0001
0.0325
<0.0001
LIHC
0.0181
0.0006
0.1129
LUAD
<0.0001
<0.0001
<0.0001
LUSC
<0.0001
<0.0001
<0.0001
OV
<0.0001
0.0023
0.7331
PAAD
<0.0001
<0.0001
<0.0001
Gene expression, normalized counts
0
5
10
15
20
LDHA
TFRC
SOD1
Normal
Tumor

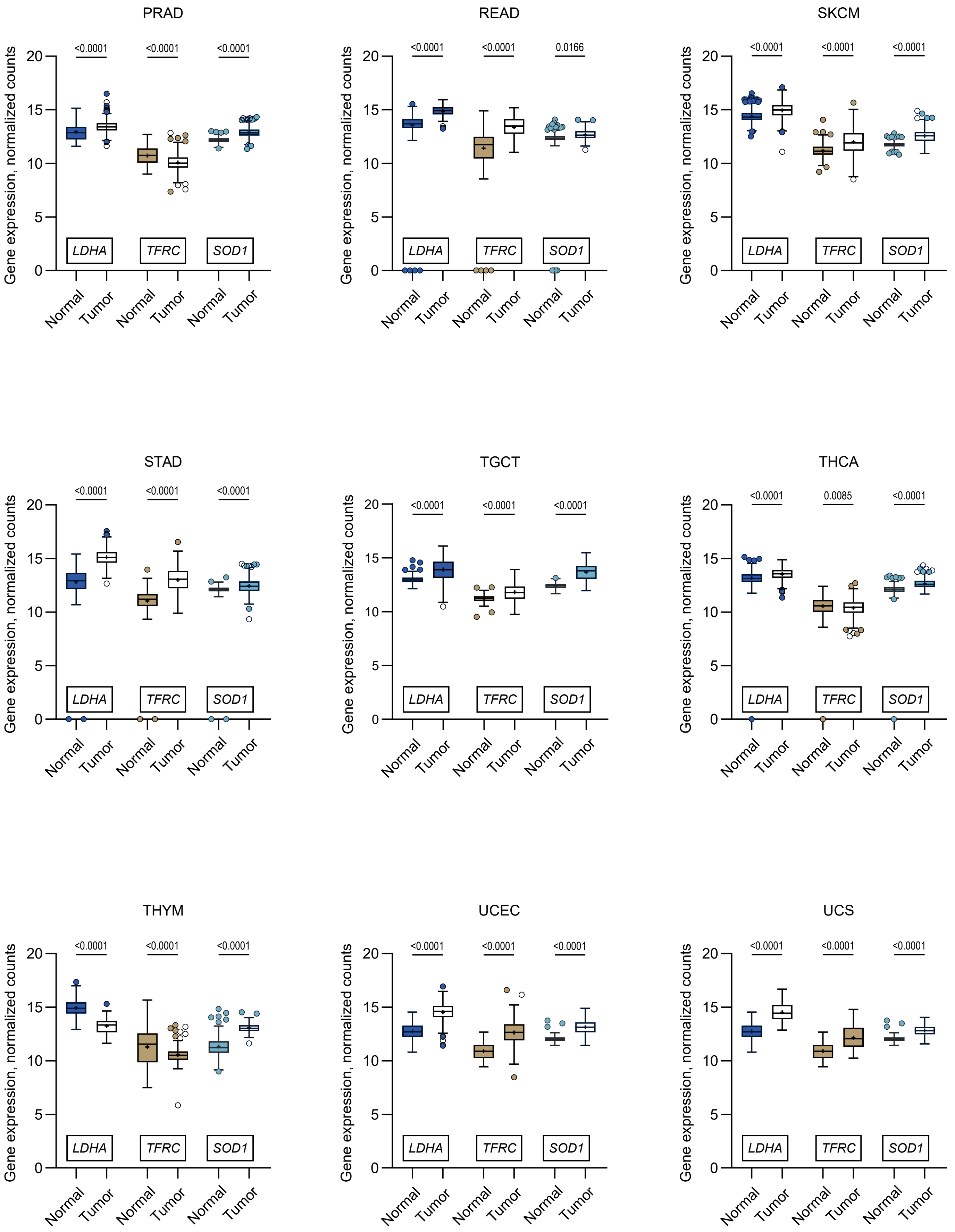
PRAD
<0.0001
<0.0001
<0.0001
Gene expression, normalized counts
20
15
10
5
0
LDHA
TFRC
SOD1
Normal
Tumor
Normal
Tumor
Normal
Tumor
READ
<0.0001
<0.0001
0.0166
Gene expression, normalized counts
LDHA
TFRC
SOD1
SKCM
<0.0001
<0.0001
<0.0001
Gene expression, normalized counts
LDHA
TFRC
SOD1
STAD
<0.0001
<0.0001
<0.0001
Gene expression, normalized counts
LDHA
TFRC
SOD1
TGCT
<0.0001
<0.0001
<0.0001
Gene expression, normalized counts
LDHA
TFRC
SOD1
THCA
<0.0001
0.0085
<0.0001
Gene expression, normalized counts
LDHA
TFRC
SOD1
THYM
<0.0001
<0.0001
<0.0001
Gene expression, normalized counts
LDHA
TFRC
SOD1
UCEC
<0.0001
<0.0001
<0.0001
Gene expression, normalized counts
LDHA
TFRC
SOD1
UCS
<0.0001
<0.0001
<0.0001
Gene expression, normalized counts
LDHA
TFRC
SOD1

Supplemental Fig. S1. Differential expression of LDHA, TFRC, and SOD1 across cancer types.
Re-computed gene expression data from TCGA and GTEx were downloaded from the UCSC Xena database [1]. Box and whiskers plots showing gene expression levels of LDHA, TFRC, and SOD1 in tumor and corresponding normal tissues for individual cancer entities from The Cancer Genome Atlas (TCGA). Normal tissues from the Genotype-Tissue Expression (GTEx) project were matched to TCGA tumor types based on organ site. P values are from 1-way ANOVA with Holm-Šídák's correction for multiple comparisons.

Sample sizes per cancer type:

TCGA	Detail	No. normal samples	No. TCGA Tumor	GTEx organ site
ACC	Adrenocortical carcinoma	128	77	Adrenal Gland
BLCA	Bladder Urothelial Carcinoma	9	407	Bladder
BRCA	Breast invasive carcinoma	179	1099	Breast
CESC	Cervical squamous cell carcinoma and endocervical adenocarcinoma	10	306	Cervix Uteri
CHOL	Cholangio carcinoma			-
COAD	Colon adenocarcinoma	308	290	Colon
DLBC	Lymphoid Neoplasm Diffuse Large B-cell Lymphoma	337	47	Blood
ESCA	Esophageal carcinoma	653	182	Esophagus
GBM	Glioblastoma multiforme	1141	166	Brain
HNSC	Head and Neck squamous cell carcinoma			-
KICH	Kidney Chromophobe	28	66	Kidney
KIRC	Kidney renal clear cell carcinoma	28	531	Kidney
KIRP	Kidney renal papillary cell carcinoma	28	289	Kidney
LAML	Acute Myeloid Leukemia	70	173	Bone Marrow
LGG	Brain Lower Grade Glioma	1141	523	Brain
LIHC	Liver hepatocellular carcinoma	110	371	Liver
LUAD	Lung adenocarcinoma	288	515	Lung
LUSC	Lung squamous cell carcinoma	288	498	Lung
MESO	Mesothelioma			-
OV	Ovarian serous cystadenocarcinoma	88	427	Ovary
PAAD	Pancreatic adenocarcinoma	167	179	Pancreas

PCPG	Pheochromocytoma and Paraganglioma			-
PRAD	Prostate adenocarcinoma	100	496	Prostate
READ	Rectum adenocarcinoma	308	93	Colon
SARC	Sarcoma			-
SKCM	Skin Cutaneous Melanoma	556	469	Skin
STAD	Stomach adenocarcinoma	174	414	Stomach
TGCT	Testicular Germ Cell Tumors	165	154	Testis
THCA	Thyroid carcinoma	279	512	Thyroid
THYM	Thymoma	337	119	Blood
UCEC	Uterine Corpus Endometrial Carcinoma	78	181	Uterus
UCS	Uterine Carcinosarcoma	78	57	Uterus
UVM	Uveal Melanoma			-

References

[1] M.J. Goldman, B. Craft, M. Hastie, K. Repečka, F. McDade, A. Kamath, A. Banerjee, Y. Luo, D. Rogers, A. Brooks, J. Zhu, C. Haussler, *Visualizing and interpreting cancer genomics data via the Xena platform*, *Nat Biotechnol* **38** (2020) 675–678. https://doi.org/10.1038/s41587-020-0546-8.
