# Supplementary material for: Raising the iron curtain: Lactate's secret role in oxidative stress defense
Source: Redox Biol. 2025 Jul 5;85:103754. doi: 10.1016/j.redox.2025.103754 (PMC12281059; doi:10.1016/j.redox.2025.103754)
Supplement: Multimedia component 2 [file mmc2.pdf]

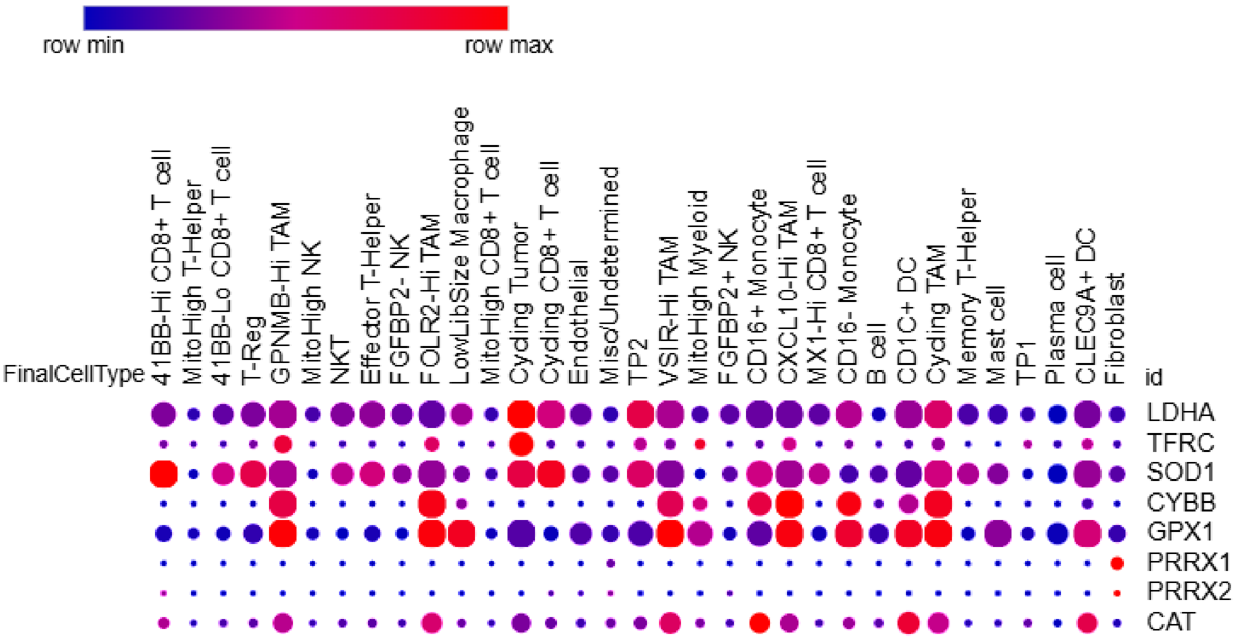

Supplemental Fig. S2. No Upregulation of H_2O_2-Detoxifying Enzymes. Catalase, peroxiredoxin 1 (Prx1), peroxiredoxin 2 (Prx2), and glutathione peroxidase 1 (GPx1) are not markedly upregulated in the 'Cycling Tumor' and 'TP2' subpopulations, despite evident H_2O_2 stress indicated by high NOX2 (CYBB) expression across all tumor-associated macrophage (TAM) populations—further supporting the idea that H_2O_2 detoxification may proceed via non-enzymatic mechanisms.
